# Light Chain (AL) Cardiac Amyloidosis: A Diagnostic Dilemma

**DOI:** 10.7759/cureus.19278

**Published:** 2021-11-05

**Authors:** Basel Abdelazeem, Nouraldeen Manasrah, Amman Yousaf, Rudin Gjeka, Arvind Kunadi

**Affiliations:** 1 Internal Medicine, McLaren Health Care, Michigan State University, Flint, USA; 2 Internal Medicine, Detroit Medical Center (DMC) Sinai-Grace Hospital, Detroit, USA; 3 Internal Medicine, McLaren Health Care, Flint, USA; 4 Cardiology, McLaren Regional Medical Center, Flint, USA

**Keywords:** case report, cardiac imagings, transthyretin, light chain, amyloidosis

## Abstract

Amyloidosis is a clinical condition characterized by amyloid fibril deposition into different organ systems. The most common types are light chain (AL) amyloidosis and transthyretin amyloidosis (ATTR) amyloidosis. Amyloidosis involves the heart with an incidence of 1.38 to 3.69 per 100,000 person-years and a prevalence of 14.85 per 100,000 person-years between 2004 and 2018. Diagnosis of cardiac amyloidosis can be made through cardiac imaging, including cardiac magnetic resonance imaging (CMR) and 99mTc-labeled pyrophosphate (PYP) cardiac scan. However, a tissue biopsy is frequently needed to confirm the diagnosis. Herein, we report such a case of cardiac amyloidosis. The patient presented with pericardial effusion and acute kidney injury as the initial presentation. The presumptive diagnosis was ATTR amyloidosis, but the endomyocardial biopsy confirmed the diagnosis of AL amyloidosis. The patient was started on bortezomib, cyclophosphamide, and dexamethasone therapy. We aimed to highlight the different diagnostic modalities of cardiac amyloidosis and the importance of obtaining tissue biopsy to confirm the amyloidosis type before starting the treatment.

## Introduction

Amyloidosis refers to the deposition of extracellular protein into different body organs. Amyloidosis has 22 different types of localized forms and 18 types of systemic forms [1]. The most common systemic forms are light chain (AL) amyloidosis and transthyretin amyloidosis (ATTR) [2]. The clinical manifestations depend on organ involvement. Cardiac amyloidosis is becoming a common condition with an incidence of 1.38 to 3.69 per 100,000 person-years and a prevalence of 14.85 per 100,000 person-years between 2004 and 2018 [3]. Cardiac amyloidosis presents with symptoms and signs of heart failure, conduction system abnormalities, and ischemia. Cardiac involvement was reported in 88% of patients with AL amyloidosis compared to 26% of patients with ATTR [4]. Our case highlights the importance of early diagnosis to improve the patients' quality of life and discusses the different diagnostic modalities of amyloidosis.

## Case presentation

A 72-years-old African American female with a past medical history of hypothyroidism presented to our hospital with dyspnea, orthopnea, paroxysmal nocturnal dyspnea, and leg swelling. The patient denied chest pain, palpitation, fever, or night sweats. The patient denied any heart disease, and she was never on any medications for heart disease. On physical exam, vital signs were blood pressure 146/96 mmHg, heart rate 91 beats per minute, 98.1 F temperature, and the patient was saturating 94% on a two-liter supplemental oxygen using a nasal cannula. The patient had distant heart sounds but regular S1 and S2, no murmurs, rubs, or gallops. The patient had an equal breathing sound bilateral, without any wheezes or ronchi. Extremities revealed bilateral 2+ edema pedal edema.

**Figure 1:**
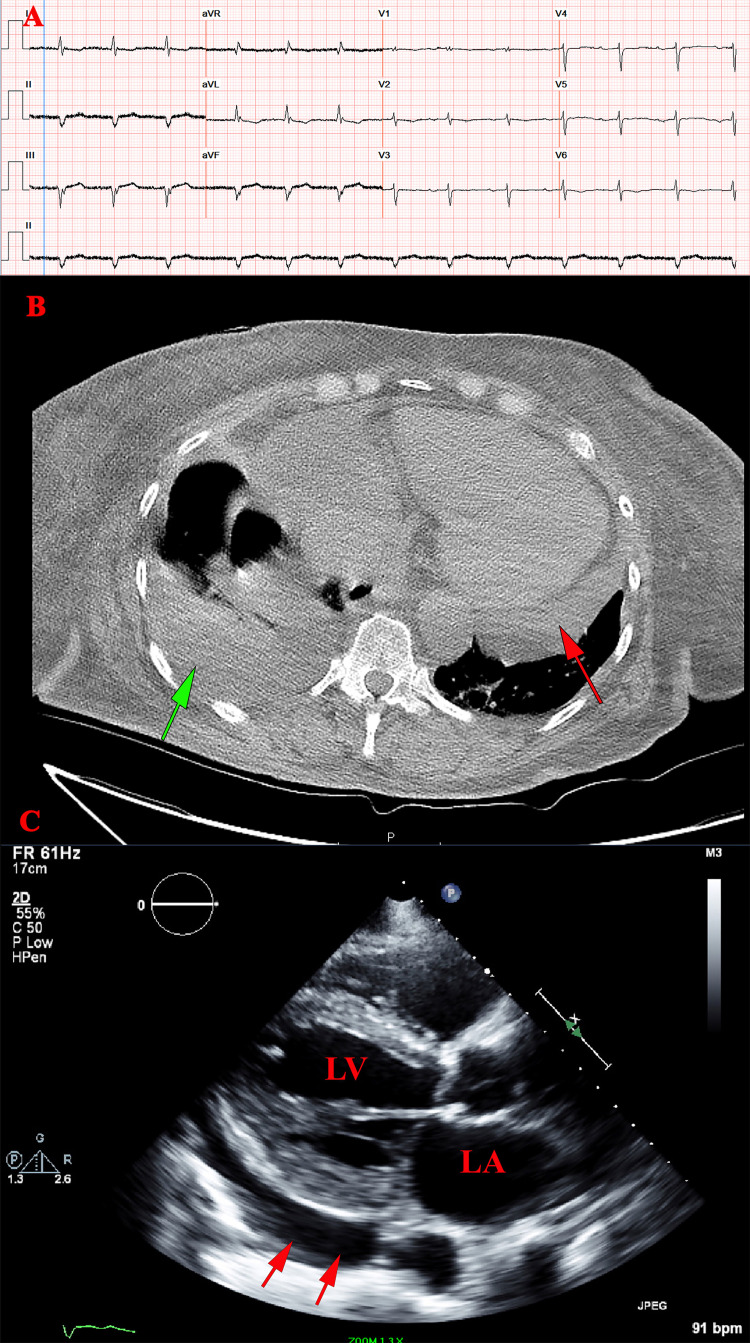
Patient's work up A) EKG revealed prolonged PR interval and type one atrioventricular block. B) Computerized tomography of the chest revealed moderate cardiomegaly, pericardial effusion, and small right pleural effusion; red arrow is pointing to pericardial effusion; the green arrow is pointing to pleural effusion. C) Transthoracic 2D echocardiogram revealed a moderate to large pericardial effusion behind LV without tamponade; arrows are pointing to the pericardial effusion. LV: left ventricle; LA: left artium.

Laboratory workup is summarized in Table 1. Electrocardiogram (EKG) revealed prolonged PR interval and type one atrioventricular block (Figure 1A). Computerized tomography of the chest revealed moderate cardiomegaly, pericardial effusion, and slight right pleural effusion (Figure 1B). An echocardiogram revealed a left ventricular (LV) ejection fraction of 25%-30%, severely increased left atrial volume 48.6 ml/m2, moderate pulmonary hypertension with right ventricular systolic pressure of 53.66 mmHg, and a moderate to large pericardial effusion behind LV without tamponade (Figure 1C) (Video 1). Cardiothoracic surgery was consulted, and the patient underwent a pericardial window with about 600 ml of fluid removed, the fluid was sent for Congo red stain, and it was negative. The cardiothoracic team placed a chest tube in the pericardial cavity which was removed later when the patient was more stable. 

**Table 1:** Laboratory workup FLC: free light chain; WBC: white blood cell; BUN: blood urea nitrogen; L: low; H: high; NA: non-applicable.

Lab	Value	References
WBC Count	3.66 L	4.50-11.00 x 10*3/uL
Hemoglobin	10.9 L	12.0-15.7 g/dL
Platelet Count	179	140-440 x 10*3/ uL
BUN	19	7-22 mg/dL
Creatinine	2.11 H	0.50-1.50 mg/dL
C Reactive Protein	4.3 H	0.0-0.8 mg/dL
BNPEP Brain Natriuretic Peptide	>5000 H	2-100 pg/mL
Troponin-I High Sensitivity	0.1880 H	0.0000-0.0400 ng/mL
Kappa FLC-Serum	2.96 H	0.33-1.94 mg/dL
Lambda FLC-Serum	9.68 H	0.57-2.63 mg/dL
Kap/Lam FLC Ratio-Ser	0.306	0.260-1.650
Serum Immunofixation	IgG lambda paraprotein	NA
Immunoglobulin IgA	175.0	60.0-350.0 mg/dL
Immunoglobulin IgG	1950.0 H	700.0-1600.0 mg/dL
Immunoglobulin IgM	46.2	40.0-280.0 mg/dL
Antineutrophil Cytoplasmic Antibodies- MPO unit	<0.2	Negative < 1.0 AI; Positive ≥ 1.0 AI
Antineutrophil Cytoplasmic Antibodies- PR3 unit	<0.2	Negative < 1.0 AI; Positive ≥ 1.0 AI
Vitamin B12	764.0	200.0-944.0 pg/mL
Erythropoietin	7.36	2.00-30.00 mIU/mL
Ferritin	372.7 H	10.0-291.0 ng/mL
Total Iron Binding Capacity	270	228-460 ug/dL

**Video 1:** Transthoracic echocardiogram Transthoracic 2D echocardiogram revealed a left ventricular ejection fraction of 25%-30% and severely increased left atrial volume 48.6 ml/m2

On telemetry, multiple pauses were noted, with the most prolonged pause of 2.5 sec and premature ventricular contractions. The patient was not able to undergo cardiac catheterization due to acute kidney injury. The patient was discharged with a plan of two-week event monitor, life vest, outpatient cardiac magnetic resonance imaging (CMR), and 99mTc-labeled pyrophosphate (PYP) cardiac scan.

**Figure 2:**
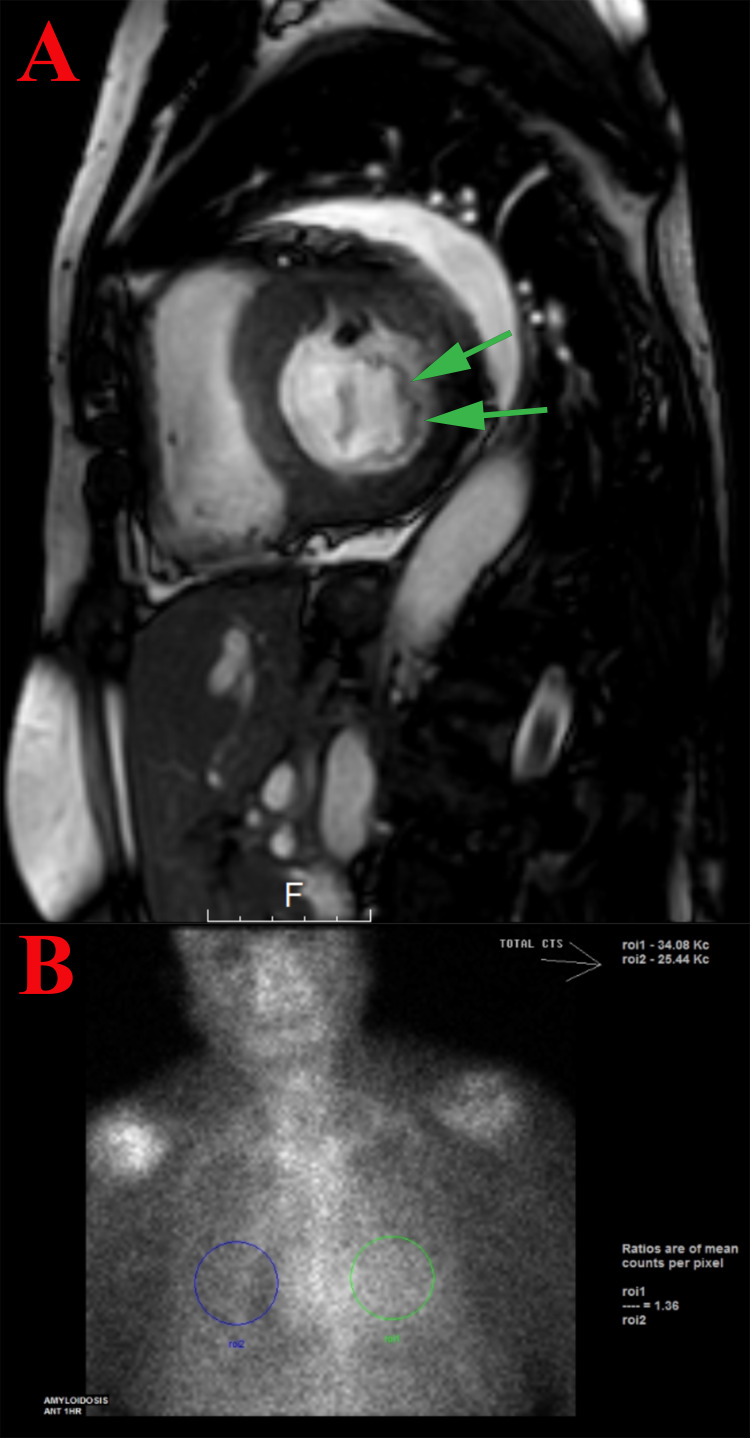
Caridiac imagings A) Cardiac magnetic resonance imaging (CMR) showed mildly increased LV thickness at the basal septal wall 13 mm with prominent trabeculations in the mid and apical segments (the green arrows). High T1 value (average 1240), which may indicate infiltrative myocardial disease. B) 99mTc-labeled pyrophosphate (PYP) cardiac scan showed features strongly suggestive of transthyretin cardiac amyloidosis with grade three of the visual interpretation (the green circle indicates the degree cardiac tissue, the blue circle indicates the degree of rib tissue).

**Figure 3:**
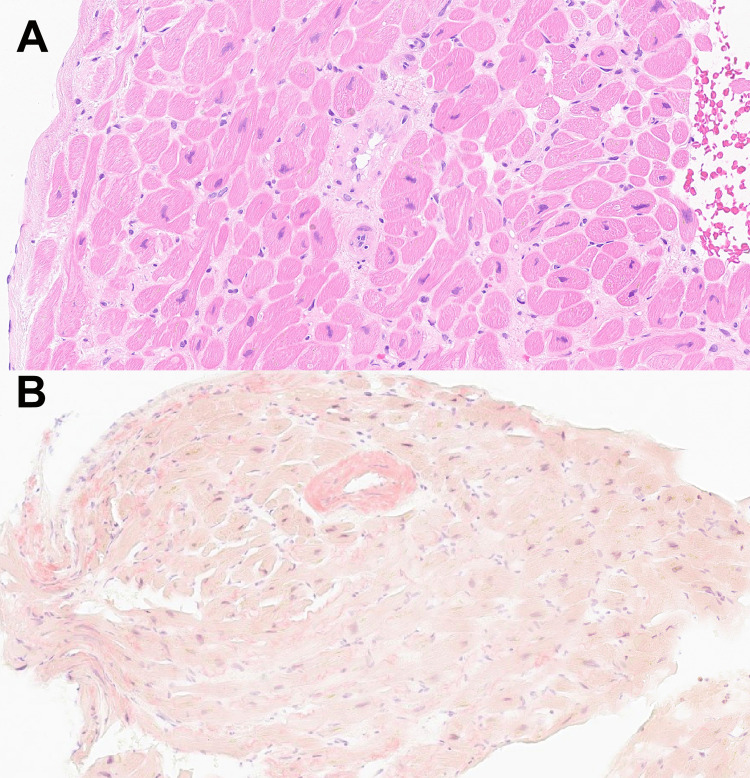
Histopathology of the endomyocardial biopsy Histopathology with 10x power showed amyloid deposits between the cardiac muscles and around the blood vessels. A) hematoxylin and eosin stain. B) Congo red stain.

CMR showed the left and right ventricles are moderately dilated. Mildly increased LV thickness at the basal septal wall 13 mm with prominent trabeculations in the mid and apical segments. The LV ejection fraction is 33%. High T1 value (average 1240), which may indicate infiltrative myocardial disease (Figure 2A). The patient underwent CT guided percutaneous core biopsy of the abdominal fat pad, and it was negative for both amyloid deposit and Congo red staining. The PYP scan was strongly suggestive of ATTR amyloidosis with grade 3 of the visual interpretation (Figure 2B). A genetic test was sent and was negative. Endomyocardial biopsy was done and revealed AL amyloidosis (Figure 3). The patient was started on bortezomib, cyclophosphamide, and dexamethasone therapy.

## Discussion

Cardiac amyloidosis presents with symptoms and signs of restrictive cardiomyopathy and right-sided heart failures, brady or tachyarrhythmia, and angina [5,6]. Extracardiac manifestations include fatigue, weight loss, peripheral neuropathy, carpal tunnel syndrome, nephrotic syndrome, macroglossia, and periorbital purpura [7].

Laboratory test abnormalities include elevated brain natriuretic peptide, troponin, and proteinuria. Monoclonal proteins testing is essential for diagnosis, and the following test should be performed serum kappa/lambda free light chain ratio, serum protein immunofixation, and urine protein immunofixation. EKG criteria include pseudo-infarction patterns, low QRS voltage, and conduction system abnormalities [8].

The echocardiogram is the initial diagnostic test of choice, and the earliest finding is relative apical sparing of longitudinal strain. Other findings include ventricular hypertrophy, thickening of the inter-atrial septum and the valves, dilated atrium, and speckled myocardium [9]. CMR is the test of choice to diagnose cardiac amyloidosis with a characteristic appearance on the late gadolinium enhancement over the entire subendocardial circumference. PYP cardiac scan has a sensitivity of 82% and specificity of 98.8% for cardiac amyloidosis if scintigraphy revealed grade 1, 2, or 3, also it helps to distinguish ATTR from AL amyloidosis [10]. The presence of grade 2 or 3 scintigraphy in patients without monoclonal protein is 100% specific for ATTR amyloidosis [11].

Our patient had the symptoms and signs of heart failure, elevated monoclonal protein, EKG finding consistent with cardiac amyloidosis, and pericardial effusion on imaging which make cardiac amyloidosis one of the differential diagnoses. However, the CMR and PYP scans were not conclusive about the diagnosis, which warranted a tissue biopsy. Although our patient had a PYP scan that was suggestive of ATTR cardiac amyloidosis, the endomyocardial biopsy revealed AL amyloidosis.

Cardiac amyloidosis has a poor prognosis, but chemotherapy, stem cell transplantation, and cardiac transplantation can improve the survival rate in a selected patient. Li et al. compared placebo versus tafamidis in ATTR cardiomyopathy patients and reported that tafamidis reduced all-cause mortality by 30% compared to placebo [12]. In contrast, The main regime for AL amyloidosis is bortezomib, cyclophosphamide, and dexamethasone. Kastritis et al. evaluated the addition of daratumumab to bortezomib, cyclophosphamide, and dexamethasone to treat AL amyloidosis. They reported increased survival and higher complete hematological response. Thus daratumumab can be considered in resistant cases; however, more studies are needed to confirm this finding [13]. Thus, a definitive diagnosis should be made before starting the treatment plan, and the management varies depending on the type of amyloidosis.

## Conclusions

Cardiac amyloidosis may be caused by AL or ATTR amyloidosis. The diagnosis of cardiac amyloidosis can be challenging through cardiac imaging, and tissue biopsy may be required to confirm the diagnosis. We present a case of cardiac amyloidosis which was presumptively diagnosed with ATTR amyloidosis depending on the PYP scan, but the endomyocardial biopsy revealed AL amyloidosis. The clinicians should consider different types of amyloidosis and reach a definitive diagnosis before starting the treatment plans.
